# Impact of Age on Long-Term Urinary Continence after Robotic-Assisted Radical Prostatectomy

**DOI:** 10.3390/medicina59061153

**Published:** 2023-06-15

**Authors:** Cristina Cano Garcia, Mike Wenzel, Clara Humke, Clarissa Wittler, Julius Dislich, Reha-Baris Incesu, Jens Köllermann, Thomas Steuber, Markus Graefen, Derya Tilki, Pierre I. Karakiewicz, Luis A. Kluth, Felix Preisser, Felix K. H. Chun, Philipp Mandel, Benedikt Hoeh

**Affiliations:** 1 Department of Urology, University Hospital Frankfurt, Goethe University Frankfurt, 60590 Frankfurt am Main, Germany; 2 Cancer Prognostics and Health Outcomes Unit, Division of Urology, University of Montréal Health Center, Montréal, QC H2X 0A9, Canada; 3 Martini-Klinik Prostate Cancer Center, University Hospital Hamburg-Eppendorf, 20251 Hamburg, Germany; 4 Dr. Senckenberg Institute of Pathology, University Hospital Frankfurt, 60590 Frankfurt am Main, Germany; 5 Department of Urology, University Hospital Hamburg-Eppendorf, 20246 Hamburg, Germany; 6 Department of Urology, Koc University Hospital, 34010 Istanbul, Turkey

**Keywords:** urinary continence, urinary incontinence, age, robotic-assisted radical prostatectomy

## Abstract

*Aim and Objectives*: We aimed to test the impact of age on long-term urinary continence (≥12 months) in patients undergoing robotic-assisted radical prostatectomy. *Methods and Materials*: We relied on an institutional tertiary-care database to identify the patients who underwent robotic-assisted radical prostatectomy between January 2014 and January 2021. Patients were divided into three age groups: age group one (≤60 years), age group two (61–69 years) and age group three (≥70 years). Multivariable logistic regression models tested the differences between the age groups in the analyses addressing long-term urinary continence after robotic-assisted radical prostatectomy. *Results*: Of the 201 prostate cancer patients treated with robotic-assisted radical prostatectomy, 49 (24%) were assigned to age group one (≤60 years), 93 (46%) to age group two (61–69 years) and 59 (29%) to age group three (≥70 years). The three age groups differed according to long-term urinary continence: 90% vs. 84% vs. 69% for, respectively, age group one vs. two vs. three (*p* = 0.018). In the multivariable logistic regression, age group one (Odds Ratio (OR) 4.73, 95% CI 1.44–18.65, *p* = 0.015) and 2 (OR 2.94; 95% CI 1.23–7.29; *p* = 0.017) were independent predictors for urinary continence, compared to age group three. *Conclusion*: Younger age, especially ≤60 years, was associated with better urinary continence after robotic-assisted radical prostatectomy. This observation is important at the point of patient education and should be discussed in informed consent.

## 1. Introduction

Prostate cancer is one of the most frequently occurring cancers for men in Europe [1]. On the one hand, more men were diagnosed with prostate cancer at an earlier age due to the use of prostate-specific antigen screening in recent years. Specifically, prostate-specific antigen screening is highly recommended for patients at age 45. A longer life expectancy for these younger patients exposes them to the potentially long-term effects of treatment-related morbidities as well as the long-term risk of disease progression leading to prostate cancer death, even though the majority of younger men with prostate cancer are diagnosed with lower-risk prostate cancer. On the other hand, due to increased life expectancy, more older patients diagnosed with prostate cancer are treated with curative treatment options. Among these treatment options, robotic-assisted radical prostatectomy is a surgical approach providing optimal oncological outcomes for clinically localized and locally advanced prostate cancer [2,3,4,5,6,7]. However, functional outcomes such as urinary continence represent an important topic, especially considering the impact of concomitant health-related quality of life for prostate cancer patients [2,8,9,10]. Urinary incontinence following robotic-assisted radical prostatectomy has been previously identified as a significant factor that negatively affects the quality of life for patients and could potentially cause substantial discomfort [2,8,9]. Ilie at al. observed a non-negligible association between urinary incontinence and increased mental distress in a contemporary prostate cancer cohort treated with radical prostatectomy [10]. Specifically, the odds ratio for experiencing mental distress was found to be 4.79 times higher among prostate cancer patients treated with radical prostatectomy who had moderate to severe urinary problems compared to those with mild urinary problems. Moreover, earlier urinary continence achievement in younger patients undergoing radical prostatectomy and consequently better rates of early urinary continence for younger patients was observed in previous analyses [11,12,13]. However, data referring to the impact of age on long-term (≥12 months) urinary incontinence are conflicting [12,13,14,15,16,17,18,19]. Moreover, fundamental surgical knowledge of prostate anatomy is crucial and new surgical techniques were developed to improve the functional outcome [20,21]. With the implementation of full functional-length urethra preservation (FFLU) and neurovascular structure-adjacent frozen-section examination (NeuroSAFE) as the new standard of care in our institution in November 2017, better rates of urinary continence could be observed [22].

The current study aimed to test the impact of age on long-term urinary continence in prostate cancer patients undergoing robotic-assisted radical prostatectomy according to different age groups. Moreover, the magnitude of the improvement rates in urinary continence due to the implementation of a new surgical standard (FFLU + NeuroSAFE) for different age groups was uncertain. We hypothesized modest differences in long-term urinary continence rates between the different age groups. Moreover, we hypothesized that modest differences existed in the improvement rates of urinary continence between different age groups due to the implementation of the new surgical standard. To address these hypotheses, we compared the long-term urinary continence rates and quantified the differences in the improvement rates of urinary continence before and after the implantation of FFLU + NeuroSAFE between the different age groups.

## 2. Materials and Methods

### 2.1. Patient Population

We used the radical prostatectomy database that our institution had prospectively collected, which to the current time point contained consecutive patients undergoing radical prostatectomy between January 2014 and January 2021. The patients in the current study period were treated by multiple surgeons that were all experienced surgeons trained in high-volume prostate cancer centers. Inclusion criteria consisted of histologically confirmed prostate cancer patients treated with robotic-assisted radical prostatectomy. Only patients with available long-term urinary continence data, defined as continence at 12 months or later (≥12 months), were included. Urinary continence information was derived from self-questionnaires that were sent to the patients, as previously described [2,23]. Moreover, urinary continence was defined by the usage of no or one safety pad within 24 h. Exclusion criteria consisted of patients that were treated with neoadjuvant chemotherapy or hormonal therapy such as testosterone-lowering therapy, anti-androgens and new androgen receptor pathway inhibitors. Moreover, patients with clinical suspicion of metastases were excluded. Starting in November 2017, the new surgical technique FFLU + NeuroSAFE was introduced in our institute. The FFLU technique is based on the identification and careful separation of the striated and smooth muscle components of the urethral sphincter located within the prostate apex and extending all the way to the colliculus. Within the NeuroSAFE technique, neurovascular bundle preservation (NVBP) is based on the intraoperative frozen section technique (IFT). If the intraoperative frozen section analysis identified positive surgical margins in the area where the neurovascular bundle was resected, it was standard practice to perform a secondary resection of the affected site. Ethical approval was obtained from the institutional review boards of the University Cancer Center Frankfurt and the Ethical Committee at the University Hospital Frankfurt, and written informed consent was obtained from all patients.

### 2.2. Statistical Analyses

Stratification was performed according to age. Age was applied as a continuous and categorical variable, on the basis of median age and interquartile range, resulting in three similarly sized groups: age group one (≤60 years) vs. age group two (61–69 years) vs. age group three (≥70 years) of 48 vs. 94 vs. 59 patients, respectively. In the subgroup analyses, we tested for age group differences within and between the two surgical techniques (standard before November 2017 and FFLU + NeuroSAFE since November 2017), considering the changes in surgical techniques to improve urinary continence [22,23]. For continuous variables, median and interquartile range (IQR) were calculated. The Kruskal–Wallis rank sum test was used to compare the medians between two groups. For categorical variables, frequencies and proportions/percentages were provided. Pearson’s chi-square test or Fisher’s exact test were used to compare the distributions in categories. Multivariable logistic regression analyses were performed to test for differences between the three age groups in analyses addressing urinary continence. Covariables consisted of surgical technique (FFLU + NeuroSAFE vs. standard), nerve-sparing status, extraprostatic tumor extension (pathological tumor stage (pT): pT2 vs. pT3/4), pathological lymph node stage (pN stage: pN0 vs. pN1 vs. pNX), surgical margin status, as well as body mass index (BMI) and prostate volume as continuous variables. The significance level was set at *p* < 0.05. For all statistical analyses, R Software Environment for Statistical Computing and Graphics (R version 4.1.3, R Foundation for Statical Computing, Vienna Austria) was used [24].

## 3. Results

### 3.1. Descriptive Characteristics

Of the 201 prostate cancer patients treated with robotic-assisted radical prostatectomy, 49 (24%) were assigned to age group one (≤60 years), 93 (46%) to age group two (61–69 years) and 59 (29%) to age group three (≥70 years). The three examined age groups differed according to the prostate volume (*p* = 0.02): Age group three presented with higher prostate volume (median volume 45 cm^3^), followed by age group two (median volume 40 cm^3^) and age group one (median volume 32 cm^3^). Moreover, the three age groups differed according to their pathological lymph node stage (pN stage; *p* < 0.001): age group one presented with the highest pN1 rates (12.5%) vs. 3% for age group two vs. 0% in age group three. Conversely, no differences between the age groups were observed according to preoperative prostate-specific antigen (PSA), body mass index, performed nerve sparing, D’Amico risk classification, extraprostatic tumor extension and positive surgical marginal status (Table 1).

### 3.2. Long-Term Urinary Continence Rates 

The overall urinary continence rate was 81%. Specifically, the three age groups differed according to long-term urinary continence: 90 vs. 84 vs. 69% for, respectively, age group one vs. two vs. three (*p* = 0.018; Table 2).

After the implementation of the new surgical technique in November 2017 (FFLU + NeuroSAFE), the overall urinary continence rate improved from 67 to 89% (Δ = 22%; *p* < 0.001). Specifically, improvement in urinary continence was highest for age group three (43 vs. 84%; Δ = 39%; *p* = 0.005), followed by age group two (74 vs. 90%; Δ = 16%; *p* = 0.09) and age group one (85 vs. 92%; Δ = 7%; *p* = 0.60; Table 3). 

### 3.3. Multivariable Logistic Regression Models 

In multivariable logistic regression, age groups one (odds ratio: 4.73, 95% confidence interval: 1.44–18.65, *p* = 0.015) and two (odds ratio: 2.94; 95% confidence interval: 1.23–7.29; *p* = 0.017) were significant predictors for urinary continence, compared to age group three (Table 4).

## 4. Discussion

This study aimed to test the impact of age on long-term urinary continence in patients undergoing robotic-assisted radical prostatectomy according to three age groups: age group one (≤60 years) vs. age group two (61–69 years) vs. age group three (≥70 years). Moreover, the magnitude of the improvement rates in urinary continence due to the implementation of a new surgical standard (FFLU + NeuroSAFE) for each of the three examined age groups is uncertain. We hypothesized modest differences in long-term urinary continence rates between the three age groups. Moreover, we hypothesized that modest differences exist in the improvement rates of urinary continence between the three age groups due to the implementation of a new surgical standard. We tested these hypotheses within our institutional database and made several important observations.

First, we observed important differences in patient and tumor characteristics between the three examined age groups. Specifically, the youngest age group, group one, exhibited the smallest prostate size (32 cm^3^), followed by age group two (40 cm^3^) and age group three (45 cm^3^, *p* < 0.002). Conversely, the youngest age group, group one, exhibited a higher rate of positive lymph nodes (12%) than age group two (3%). Interestingly, no patient presented positive lymph nodes (0%) in age group three. No differences between these three age groups were observed in PSA, BMI, D’Amico risk classification, nerve-sparing status, performed FFLU + NeuroSAFE, extraprostatic disease and positive surgical margins status. In consequence, all the above-mentioned differences may influence urinary continence outcomes after robotic-assisted radical prostatectomy in the current study. Therefore, it is of crucial importance to include these patient and tumor characteristics in multivariable analyses addressing postoperative urinary continence, as was carried out in the current analyses.

Second, we recorded important differences in the long-term urinary continence rates between the three examined age groups. Specifically, the youngest age group, group one (≤60 years) presented the highest long-term urinary continence rates (90%), followed by age group two (84%) and age group three (69%). Moreover, in multivariable logistic regression models adjusting for the above-mentioned patient and tumor characteristics, age group one (Odds Ratio 4.73, *p* = 0.015) and age group two (Odds Ratio 2.94; *p* = 0.017) represented predictors for long-term urinary continence. Overall, the data regarding the impact of age on long-term (≥12 months) urinary continence were heterogeneous in previous studies [11,12,13,14,15,16,17,18,19]. The results of the current study agreed with previous analyses observing younger patients experiencing favorable long-term urinary continence rates compared to their older counterparts [14,15,17,18,19]. Conversely, in other analyses, significant differences in long-term urinary continence rates between different age groups could not be observed [11,12,13,16,25]. It is noteworthy to mention that a heterogenous definition for urinary continence and pronounced differences in age group intervals exists. Therefore, these previous findings regarding the association between urinary continence and age in prostate cancer patients cannot be directly compared to the results of the current study. 

Third, we observed important differences in long-term urinary continence rates before and after the implementation of a new surgical technique (FFLU + NeuroSAFE, 67 vs. 89%; Δ = 22%; *p* < 0.001). Specifically, the improvement was highest for age group three (45 vs. 84%; Δ = 39%; *p* = 0.005), followed by age group two (74 vs. 90%; Δ = 16%; *p* = 0.009) and age group one (85 vs. 92%; Δ = 7%; *p* = 0.60). Schlomm et al. reported significantly increased early urinary continence results regarding FFLU surgical technique [20]. The findings of the current study indicated that the implementation of FFLU + NeuroSAFE had a substantial effect on the already good long-term urinary continence rates of the younger age group one. Moreover, in absolute numbers, the older patients in our cohort profited the most from this implementation. 

Taken together, we made important observations according to the impact of age on long-term urinary continence in robotic-assisted radical prostatectomy patients. Specifically, patients ≤60 years (age group one) experienced the highest rates of long-term urinary continence after robotic-assisted radical prostatectomy (90%). The rates even improved with the implementation of a new surgical technique (FFLU + NeuroSAFE) and yielded a long-term urinary continence rate of 92% (previously 85%). Moreover, in multivariable logistic regression models, age group one represented the strongest predictor for long-term urinary continence (Odds Ratio 4.73; *p* = 0.015). These observations are important at the point of patient education and should be discussed in informed consent. Urinary incontinence and the usage of pads can affect the quality of life in all age groups. Gondoputro et al. observed higher rates of moderate/big bother in younger patients (<55 years) when using >1 pad/24 h, compared to older patients [18]. Moreover, for younger men, a greater social stigma for pad usage could result in even more increased concomitant anxiety [26,27]. In line with these observations, Ilie et al. identified older age as a protective factor for screening positive for mental distress in prostate cancer patients treated with radical prostatectomy. On the one hand, the current results can help to overcome the preconception that radical prostatectomy leads automatically to urinary incontinence. On the other hand, functional outcomes in elderly patients have improved significantly with the implementation of new surgical approaches. Nevertheless, patients should be educated about the impact of age on long-term urinary continence. These results confirmed that a surgical approach in well-selected older patients can also provide good urinary continence rates. 

The current study is not devoid of limitations. First, this study was of retrospective nature. Therefore, the currently used data may be constrained by the possibility of bias and inaccuracies. Second, after applying the above-mentioned inclusion and exclusion criteria, the study court consisted of 201 patients. This limited sample size may restrict the generalizability of the findings to a broader population. Third, data regarding urinary continence were obtained from voluntary self-questionnaires. Therefore, the risk of selection and non-response bias should be considered. Fourth, adjustment for other variables such as relevant comorbidities that could have influenced urinary continence was not possible due to missing information. Fifth, all patients were professionally instructed in pelvic-floor training during their in-patient stay and continuing this training by seeking professional help was strongly recommended. Nevertheless, a potential bias regarding the extent of postsurgical pelvic-floor training cannot be ruled out. Moreover, variability in the available pelvic-floor training during the coronavirus disease of 2019 (COVID-19) pandemic could have influenced the urinary continence rates. Sixth and finally, robotic-assisted radical prostatectomy was performed by several surgeons over the study period. Although all surgeons were experienced and trained in high-volume prostate cancer centers, differences in the experience level among the surgeons might have been present and therefore influenced the functional outcomes. 

## 5. Conclusions

In conclusion, our study findings highlight an important correlation between age and urinary continence outcomes following robotic-assisted radical prostatectomy. Specifically, we observed that younger patients, particularly those aged 60 years or below, had better urinary continence postoperatively. This observation carries significant implications in terms of patient education and informed consent. By discussing this association with patients, healthcare providers can provide realistic expectations regarding urinary continence recovery based on age. Furthermore, emphasizing this relationship during the informed consent process allows patients to actively participate in their treatment decisions. By understanding that age plays a role in urinary continence recovery, patients can have more realistic expectations, which may lead to increased satisfaction with surgical outcomes. It also enables healthcare providers to tailor preoperative counseling and postoperative support based on age, potentially implementing additional measures or interventions to optimize continence outcomes in older patients.

## Figures and Tables

**Table 1:** Descriptive characteristics of 201 prostate cancer patients undergoing robotic-assisted radical prostatectomy (RARP) and available long-term urinary continence information between January 2014 and January 2021, according to age group.

Characteristic	Age Group 1 (≤60) ^1^ n = 49 (24%)	Age Group 2 (61–69) ^1^ n = 93 (46%)	Age Group 3 (≥70) ^1^ n = 59 (29%)	*p*-Value ^2^
PSA in mg/ml	6.7 (5.2, 9.3)	6.7 (5.2, 9.6)	9.0 (5.7, 11.9)	0.07
BMI in kg/m^2^	26.4 (25.1, 28.6)	26.6 (24.3, 29.9)	25.6 (24.2, 27.8)	0.3
Prostate volume in cm^3^	32 (27, 46)	40 (30, 47)	45 (30, 62)	0.02
Gleason grade group Biopsy-specimen				
I	15 (31%)	19 (20%)	17 (29%)	
II	21 (43%)	48 (52%)	23 (39%)	
III	8 (16%)	12 (13%)	12 (20%)	
IV	4 (8%)	10 (11%)	3 (5%)	
V	1 (2%)	4 (4%)	4 (7%)	
D’Amico risk classification				0.4
low	13 (27%)	14 (15%)	8 (14%)	
intermediate	26 (54%)	59 (64%)	40 (68%)	
high	9 (19%)	19 (21%)	11 (18%)	
Nerve sparing performed	45 (92%)	79 (86%)	48 (83%)	0.4
FFLU + NeuroSAFE performed	36 (73%)	58 (62%)	37 (63%)	0.4
Gleason grade group RARP-specimen				
I	12 (25%)	19 (20%)	9 (15%)	
II	25 (51%)	53 (57%)	25 (42%)	
III	7 (14%)	9 (10%)	15 (25%)	
IV	1 (2%)	3 (3%)	5 (9%)	
V	4 (8%)	9 (10%)	5 (9%)	
Extraprostatic tumor extension (T3/4)	15 (31%)	41 (44%)	19 (32%)	0.2
pN-stage				<0.001
pN0	37 (76%)	83 (89%)	59 (100%)	
pN1	6 (12%)	3 (3%)	0 (0%)	
pNx	6 (12%)	7 (8%)	0 (0%)	
Positive surgical margin (R1)	11 (22%)	26 (28%)	16 (27%)	0.6

^1^ Median (IQR = interquartile range); n (%), ^2^ Kruskal–Wallis rank sum test; Pearson’s chi-square test; Fisher’s exact test, Abbreviations: PSA = prostate-specific antigen, BMI = Body mass index, FFLU = full functional-length urethral sphincter preservation, NeuroSAFE = neurovascular structure-adjacent frozen-section examination; pN stage = pathological lymph node stage.

**Table 2:** Long-term urinary continence rate of 201 prostate cancer patients undergoing robotic-assisted radical prostatectomy (RARP) between January 2014 and January 2021, according to age-group.

Characteristic	Overall n = 201 (100%)	Age Group 1 (≤60) ^1^ n = 49 (24%)	Age Group 2 (61–69) ^1^ n = 94 (46%)	Age Group 3 (≥70) ^1^ n = 60 (30%)	*p*-Value ^2^
Long-term urinary continence					0.018
Yes	163 (81%)	44 (90%)	78 (84%)	42 (69%)	
No	38 (19%)	5 (10%)	16 (16%)	18 (31%)	

^1^ Median (IQR = interquartile range); n (%), ^2^ Kruskal–Wallis rank sum test; Pearson’s chi-square test; Fisher’s exact test.

**Table 3:** Comparison of long-term urinary continence rates for prostate cancer patients undergoing radical prostatectomy (RARP) according to age group between eras: before November 2017 (=standard) and since November 2017 (=FFLU + NeuroSAFE).

	Overall	Age Group 1 (≤60 Years)	Age Group 2 (61–69 Years)	Age Group 3 (≥70 Years)
FFLU + NeuroSAFE ^1^	116 (89%)	33 (92%)	52 (90%)	31 (84%)
Standard ^1^	47 (67%)	11 (85%)	26 (74%)	10 (45%)
*p*-value ^2^	<0.001	0.60	0.09	0.005

^1^ n (%), ^2^ Kruskal–Wallis rank sum test; Pearson’s chi-square test; Fisher’s exact test. Abbreviations: FFLU = full functional-length urethral sphincter preservation; NeuroSAFE = neurovascular structure-adjacent frozen-section examination.

**Table 4:** Multivariable * logistic regression models predicting long-term (≥ 12 months) urinary continence ** in 201 prostate cancer patients treated with robotic-assisted radical prostatectomy (RARP).

	Multivariable		
	Odds ratio	95% confidence interval	*p*-value
Age groups			
Age group 3 (≥70 years)	Reference	–	–
Age group 2 (61–69 years)	2.94	1.23–7.29	0.017
Age group 1 (≤60 years)	4.73	1.44–18.65	0.015

* adjusted for surgical technique (FFLU + NeuroSAFE vs. standard), nerve-sparing status, extraprostatic tumor extension (pT2 vs. pT3/4), pN stage, as well as BMI and prostate volume as continuous variables. ** Urinary continence was defined by usage of no or one safety pad within 24 h. Abbreviations: FFLU = full functional-length urethral sphincter preservation; NeuroSAFE = neurovascular structure-adjacent frozen-section examination; pN stage = pathological lymph node stage; BMI = body mass index.

## Data Availability

All data generated or analyzed during this study are included in this article. Further enquiries can be directed to the corresponding author.

## Abbreviations

BMI	body mass index
FFLU	full functional-length urethra preservation
IFT	intraoperative frozen section analysis
IQR	interquartile range
NeuroSAFE	neurovascular structure-adjacent frozen-section
NVBP	neurovascular bundle preservation
OR	odds ratio
pN stage	pathological lymph node stage
PSA	prostate-specific antigen
pT	pathological tumor stage
RARP	robotic-assisted radical prostatectomy
R1	positive surgical margin
